# Explicit access to detailed representations of feature distributions

**DOI:** 10.3758/s13423-025-02716-3

**Published:** 2025-06-06

**Authors:** Vladislav Khvostov, Árni Gunnar Ásgeirsson, Árni Kristjánsson

**Affiliations:** 1 Department of Psychology, The Ohio State University, Columbus, OH 43210 USA; 2 Icelandic Vision Lab, University of Iceland, Reykjavík, Iceland; 3 University of Akureyri, Akureyri, Iceland

**Keywords:** Ensemble perception, Feature distribution representations, Perceptual organization, Set perception

## Abstract

The human visual system can quickly process groups of objects (ensembles) and build compressed representations of their features. What does the conscious perception of ensembles consist of? Observersʼ explicit access to ensemble representations has been considered very limited – any distributional aspects beyond simple summary statistics, such as the mean or variance, cannot be explicitly accessed. In contrast, we demonstrate that the visual system can represent ensemble distributions in detail, and observers have reliable explicit access to these representations. In our new paradigm (*Feature Frequency Report*), observers viewed 36 disks of various colors for 800 ms and then reported the frequency of a randomly chosen color using a slider. The sets had Gaussian, uniform, or bimodal color distributions with a random mean color. The distributions of responses – both aggregated and separate for each observer – followed the shape of the presented distribution. Modeling revealed that performance reflected integrated information from the whole set rather than sub-sampling. After only brief exposure to a color set**,** the visual system can build detailed representations of feature distributions that observers have explicit access to. This result necessitates a fundamental rethinking of how ensembles are processed. We suggest that such distribution representations are the most natural way for the visual system to represent groups of objects. Explicit feature distribution representations may contribute to people ‘s impression of having a rich perceptual experience despite severe attentional and working memory limitations.

## Introduction

**Fig. 1:**
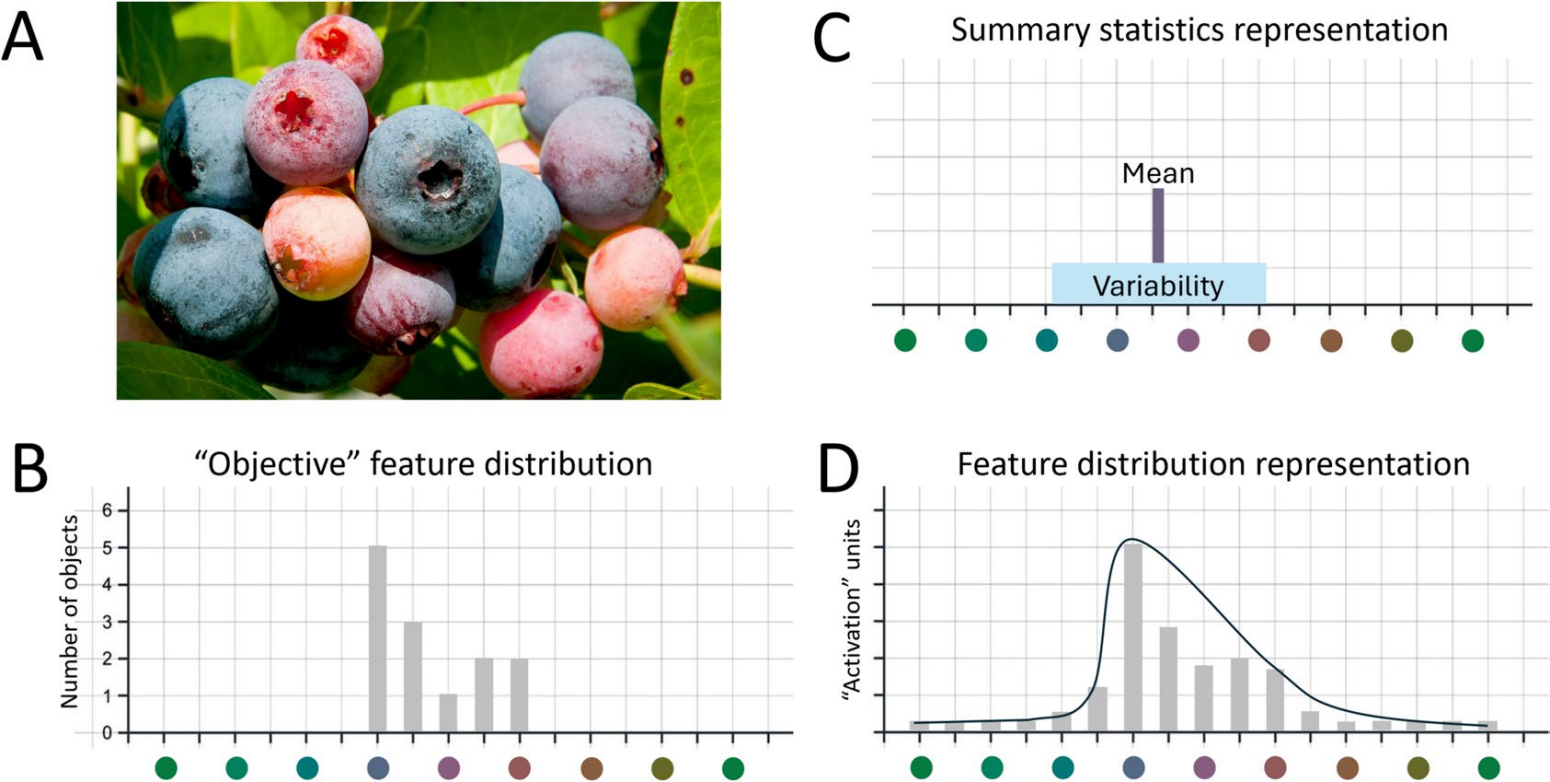
(**A, B**) A blueberry branch and its corresponding “probability density function” depicting the number of exemplars of each color. (**C**) The schematic prediction of the *Summary statistics* view suggesting that the visual system explicitly represents a set of objects as its mean and variability around it. (**D**) Schematic prediction of the *Feature distribution* view that argues for the explicit feature distribution representation of a set of objects (y-axis depicts arbitrary"activation"units)

How does the visual system represent the group of berries in Fig. 1A? What does our conscious perception of this set consist of? What can we explicitly access? These questions have been thoroughly investigated in the last 30 years in studies of ensemble perception (for reviews, see Alvarez, 2011; Corbett et al., 2023; Whitney & Yamanashi Leib, 2018). Representing a group of objects is challenging for the visual system considering its limited capacity for attending to objects and storing them in working memory for detailed processing (Cowan, 2001; Luck & Vogel, 1997; Pylyshyn & Storm, 1988). Indeed, participants perform poorly when asked about individual members of ensembles but can surprisingly accurately report the average of many visual features (Ariely, 2001; Chong & Treisman, 2003, 2005b) and estimates of their variability (e.g., range or variance: Haberman et al., 2015; Khvostov & Utochkin, 2019; Morgan et al., 2008). A popular view in the field is that the conscious (explicit) representation of an ensemble contains compressed information: instead of storing all individual exemplars, only summary statistics – mean and variance – are explicitly represented (Alvarez, 2011; Atchley & Andersen, 1995; Dakin & Watt, 1997; Hansmann-Roth et al., 2021). We refer to this as the *Summary statistics* view*.*

However, our subjective experience of viewing Fig. 1A suggests that we represent and can report much more than just the average and variance of the berry ensemble. The *Summary statistics* view suggests this is illusory. Conversely, we argue that this feeling exists because our explicit representations are much richer than just mean and variability. We propose the *Feature distribution* view: our conscious (explicit) representation of ensembles includes whole feature distributions. This representation is a noisy summary of individual feature values (Fig. 1D, cf. the “objective” distribution on 1B) that lacks detailed spatial information (which feature values are where). The distributional representation can still be used to derive and report the summary statistics if required (e.g., the distribution peak can be reported as the mean) but contains much more details: we can, for example, explicitly report that the scene in Fig. 1A contains approximately twice as many ripe dark-blue berries as unripe red-yellow ones.

Recently, Utochkin and colleagues (2024) proposed a biologically plausible model based on spatial pooling and population coding mechanisms that can explain the origin of such distributional representations. There is recent empirical evidence for the existence of feature distributional representations per se (Chetverikov et al., 2016, 2017b; Kim & Chong, 2020) and how the visual system uses them to perform various cognitive tasks (Khvostov et al., 2021; Utochkin, 2015; Utochkin & Yurevich, 2016; Utochkin et al., 2018, 2020). However, these studies involved implicit tasks where participants do not need to explicitly report feature distributions – the distribution representations are inferred indirectly (e.g., from response time (RT) patterns). Moreover, from participants’ poor ability to distinguish visual displays containing different distributions, many concluded that observers cannot explicitly report any aspects of distribution representations beyond simple summary statistics (Atchley & Andersen, 1995; Dakin & Watt, 1997; Hansmann-Roth et al., 2021).

Instead of using indirect tests, we designed a new experimental paradigm (*Feature Frequency Report* – *FFR*) that is specifically aimed at testing the explicit distributional representation. In contrast with the *Summary statistics* view, our results demonstrate that not only can the visual system build representations of the feature distributions of ensembles but, most importantly, observers have reliable explicit access to them.

## Methods

### Participants

Ten naïve volunteers participated (average age – 24.5 years, six females, four males). The sample size was based on previous studies using segmented regression for the analysis of feature distribution representations (Chetverikov et al., 2016, 2017b). Power analysis using G-power software 3.0.10 (Faul et al., 2007) revealed that ten observers gave us the power (1-β) = 0.8 (with α = 0.05) to detect effect sizes equal to Cohen’s *d* = 0.85 for a one-sample *t*-test comparing individual slopes from segmented regression. Participants had normal or corrected-to-normal visual acuity and no color vision deficiency as assessed by the Ishihara color blindness test. Participants gave written informed consent. The experiment was run following the Research Ethics Committee for Public Higher Education principles and the Declaration of Helsinki.

*Apparatus and stimuli.* Stimuli were displayed on a 24-in. calibrated LCD monitor (ASUS, VX248 h; resolution 1,920 × 1,080) using PsychoPy 3 (Peirce et al., 2019) version 2022.1.4 on a desktop PC with Windows 10. The screen was color-calibrated using a Cambridge Research Systems ColorCal MK2 photometer. The stimuli appeared within a 21.8° × 21.8° square at the center of the screen that was divided into 6 × 6 = 36 cells by an imaginary grid with a cell side of 3.85°. Each cell contained one disk. Within the cell, the positions of all disks were randomly jittered horizontally and vertically in a 0.55° range before each trial. The stimuli were colored disks with a diameter of 1.93°. We used the color wheel with 48 hues: neighboring colors are separated from each other approximately by 1 JND (just noticeable difference) (the color space was based on Witzel & Gegenfurtner, 2013; see, e.g., Chetverikov et al., 2017b; Hansmann-Roth et al., 2021).

**Fig. 2:**
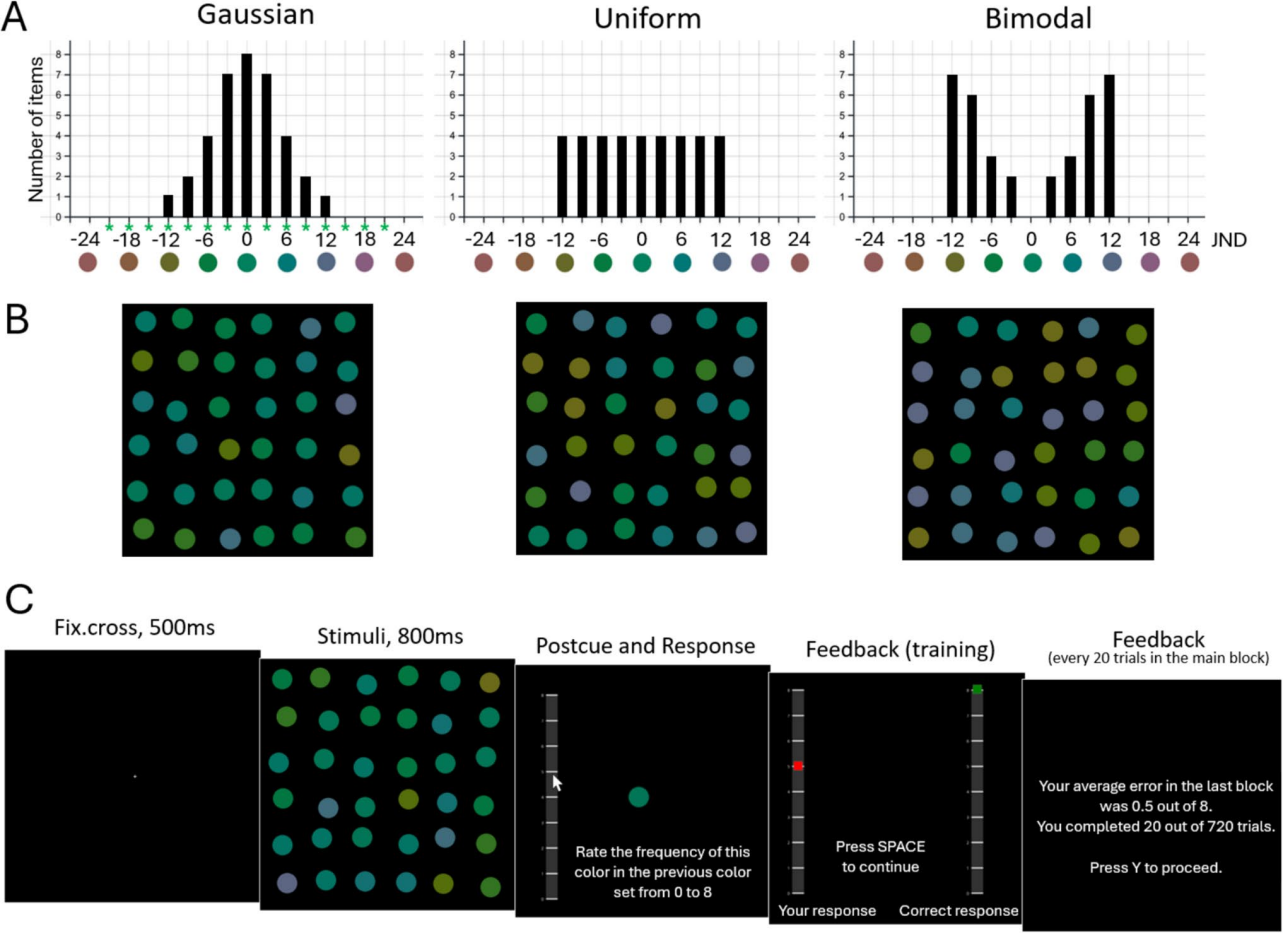
Stimuli and procedure of the experiment. (**A**): To generate a certain distribution shape, we did not draw random values from this distribution – we presented a fixed number of the color exemplars (shown here) to ensure that each trial display truly reflected a certain distributional shape (the mean color was chosen randomly for each trial). The green asterisk below the x-axis of the Gaussian distribution shows which values were tested during the experiment (the same test values were used for the other two distributions). (**B**): The corresponding examples of stimuli displays. (**C**): An example display sequence for an experimental trial. The text in the displays was enlarged for illustrative purposes

In each trial, we chose a random mean and presented 36 disks whose colors formed one of three fixed distributional shapes around this mean color value. To generate a certain distribution shape, we did not draw random values from this distribution – we presented a fixed number of the color exemplars (Fig. 2A) to ensure that each trial display truly reflected a certain distributional shape. The Gaussian distribution always contained 8 disks of the average color, 7 × 2 disks with a color that was ± 3 JND from the average (all distributions were symmetrical – i.e., seven disks with the color 3 JND from the mean in the clockwise direction, and seven disks with the color 3 JND in the counter-clockwise direction), 4 × 2 disks with ± 6 JND from the mean, 2 × 2 disks with ± 9 JND, and 1 × 2 disks with ± 12 JND. The uniform distribution contained the same colors, but each color value had four exemplars. The bimodal distribution contained no disks with the mean color, 2 × 2 disks with ± 3 JND, 3 × 2 disks with ± 6 JND, 6 × 2 disks with ± 9 JND, and 7 × 2 disks with ± 12 JND. All three distributional conditions were intermixed within a single experimental block.

### Procedure

We developed a new experimental paradigm – *Feature Frequency Report* (FFR) – to test whether the visual system has explicit access to the properties of feature distributions. Observers were briefly presented (800 ms) with ensembles of 36 colored disks from Gaussian, uniform, or bimodal distributions with a random mean color in each trial (Fig. 2A, B). They reported the frequency of a randomly chosen postcued color, from 0 to 8, using a slider. During pre-experimental training (80 trials), they received feedback about the correct response while in the main session, there was no trial-by-trial feedback – after every 20 trials, participants were presented with feedback about their average error in the last 20 trials and could take a break before proceeding (see an example trial sequence in Fig. 2C). Participants were encouraged to form a general impression of all colors in the display and give approximate responses based on this, instead of trying to count disks of different colors.

The tested color value could be the same as one of the just presented colors (0, ± 3, ± 6, ± 9, ± 12 JND from the mean) or outside the range (we tested three equally spaced values from each side of the distribution: ± 15, ± 18, ± 21 JND – green asterisks below the x-axis in Fig. 2A depict all tested values in the experiment). Probing these values and combining participants ‘ responses allowed us to evaluate directly whether feature distributions were represented explicitly. All three distributional shapes were symmetric relative to the mean, which allowed us to increase measurement reliability by folding each distribution in half: we averaged all observers’ responses from the same absolute tested color value (i.e., the same distance to the mean from both sides of the distribution).

## Results

**Fig. 3:**
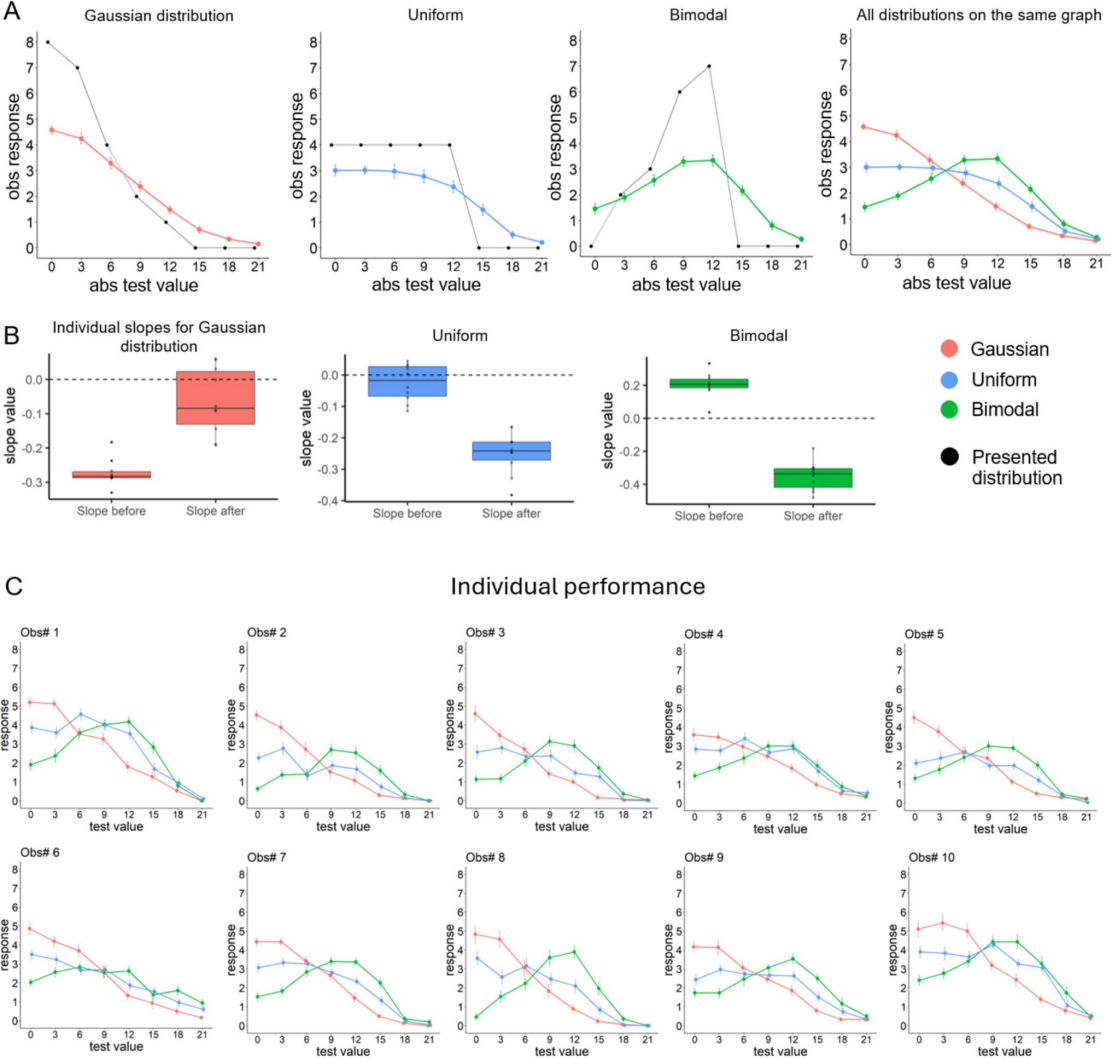
(**A**) The Gaussian, uniform, and bimodal distributions, as well as a combined version of all three curves on the same graph for comparison. Error bars denote the SEM. Colored points represent averaged observers’ slider responses as a function of the absolute tested value in a trial (“0” is the mean of the presented distribution, and “12” is the edge). Black points depict the “ground truth”: the actual number of items of each color presented. (**B**) The slope values from individual segmented regression. (**C**) The shapes of the three distributions can be seen in the individual graphs for all ten participants. Error bars denote the SEM

All recorded data were included in the analyses described below. We found that observers could precisely determine and report the presented distribution: observers’ response curves followed the shapes of the presented distributions (Fig. 3). These results provide strong evidence for the *Feature distribution* view of ensemble perception but contradict the *Summary statistics* view. Since all three distribution types had the same number of items and the same color range, the *Summary statistics* view predicts that the tested values equal to the mean should always be reported with the highest rating. However, following the prediction of the *Feature distribution* view, this is true only for the Gaussian distribution (*t*s(9) > 2.39, *p*s < 0.04, Cohen’s *d*s > 0.75), there are no such differences for the Uniform distribution (*t*s(9) < 1.6, *p*s > 0.14, Cohen’s *d*s < 0.51, except the difference between the mean and 12 JND (the edge of distribution): *t*(9) = 3.29, *p* = 0.009, Cohen’s *d* = 1.04). The Bimodal condition even produced the reverse trend (*t*s(9) < −4.47, *p*s < 0.002, Cohen’s *d*s > 1.41).

A series of segmented regressions (Muggeo, 2008) shows that observers' response curves have the same shapes as the presented distributions as predicted by the *Feature distribution* view. For the uniform distribution, average responses were stable across the presented range and monotonically decreased for values outside the range (the further from the mean, the smaller the response values). The segmented regression shows that the response curve can be represented as a two-segment linear function with a break point at 9.74 JND away from the mean (95% Confidence Intervals (CI) = [7.18, 12.3]). The slope before the break point was indistinguishable from zero (b = −0.02, 95% CI = [−0.08, 0.03]), while the slope after the break point was strongly negative (b = −0.25, 95% CI = [−0.31, −0.19]). A Davies test comparing the models with and without this break point showed a significant difference (*p* < 0.001).

The curve of the averaged responses in the Gaussian condition had different dynamics: a monotonic decrease from the mean to the edge of the distribution, which continued with a flat curve outside the range. We found a significant break point at 16.01 JND from the mean (95% CI = [13, 19.02], Davies’s *p* < 0.001). The slope was negative for the first segment (b = −0.27, 95% CI = [−0.29, −0.24]) but around zero for the second segment (b = −0.06, 95% CI = [−0.21, 0.08]).

In contrast, the bimodal distribution led to a monotonic increase of response values from the mean to the edge of the distribution with a decreasing part outside the range. The break point was at 10.88 JND (95% CI = [9.95, 11.82], Davies’s *p* < 0.001). The slope before and after the break point was positive (b = 0.21, 95% CI = [0.15, 0.26]) and negative correspondingly (b = −0.35, 95% CI = [−0.4, −0.3]).

The three presented distributions had the same number of items, range of color values, and mean, but the shapes of the distributions of observers'responses are completely different (Fig. 3A). Most importantly, these differences correspond to the objectively presented distributions: flat uniform distribution, Gaussian distribution with a peak at the mean, and a bimodal distribution with two peaks at the edges.

The segmented regression results were not an artifact of data aggregation since we also fitted models for individual observers with the break points obtained from a segmented model built on the aggregated data and compared slopes before and after the break point with zero. We perfectly replicated our results described above: in the Uniform condition, the break point at 9.74 JND divided curves into a flat segment (*t*(9) = −1.32, *p* = 0.218, Cohen’s *d* = 0.42) and a negative segment (*t*(9) = −11.53, *p* < 0.001, Cohen’s *d* = 3.65). The break point at 16.01 JND in the Gaussian condition divided the curves into negative (*t*(9) = −22.21, *p* < 0.001, Cohen’s *d* = 7.02) and flat segments (*t*(9) = −2.08, *p* = 0.067, Cohen’s *d* = 0.66). In the Bimodal condition, the break point at 9.74 JND divided the curves into positive (*t*(9) = 8.64, *p* < 0.001, Cohen’s *d* = 2.73) and negative slope segments (*t*(9) = −12.65, *p* < 0.001, Cohen’s *d* = 4). Importantly, all observers had the same sign for all corresponding slopes, except flat ones (see Fig. 3B), which shows how consistent the effects are (see Fig. 3C).

### Observers integrate information from whole sets rather than sub-sampling

**Fig. 4:**
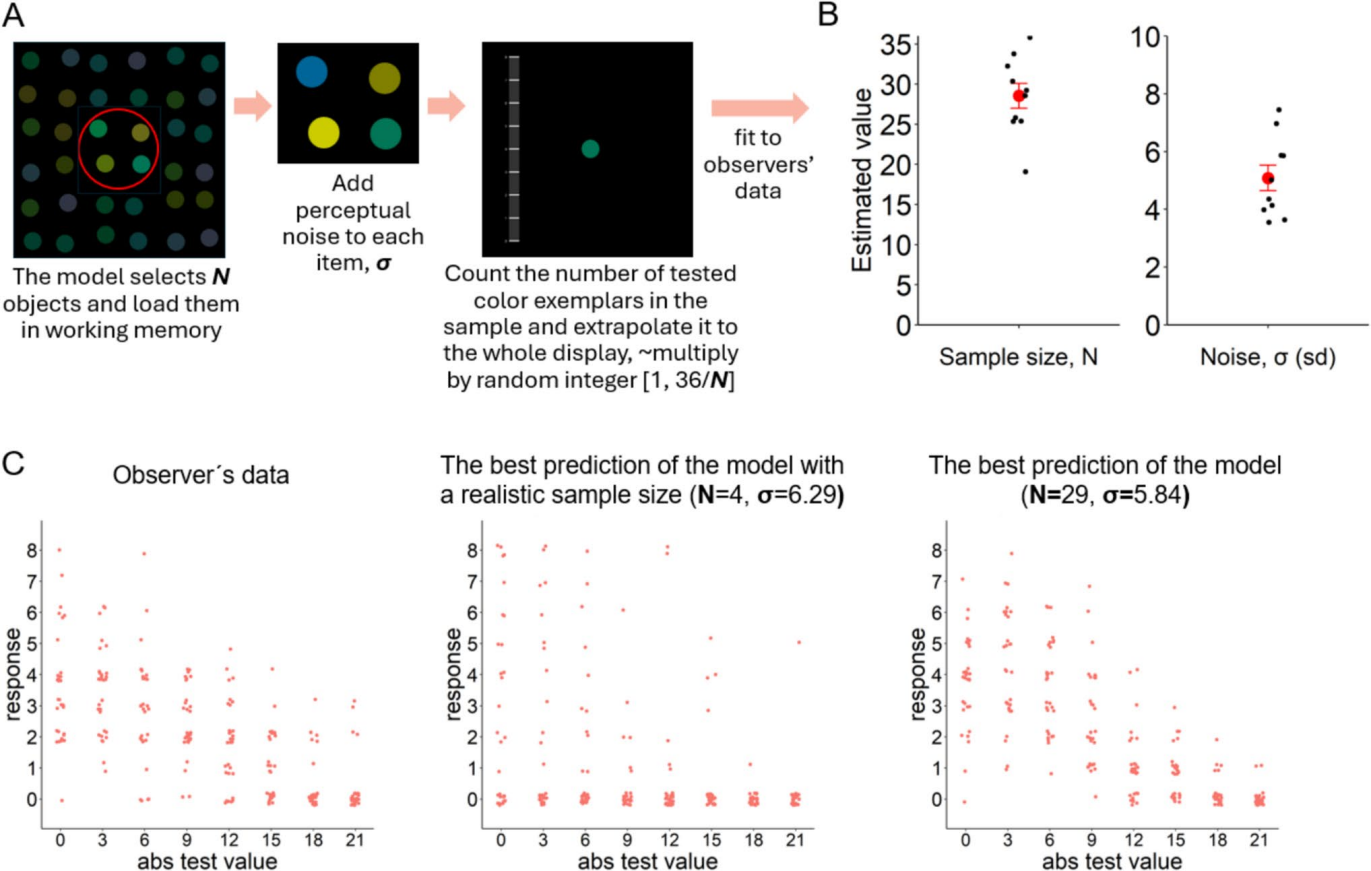
(**A**) Schematic version of the sub-sampling model. (**B**) Individual model fits for each observer. The red dots depict the means (N = 28.5, σ = 5.08) with 95% CI around it. (**C**) From left to right: All responses of an illustrative observer in the Gaussian condition along with the best prediction of the model fitted with the restriction of realistic sample sizes for working memory capacity, i.e. <  = 4 disks (**N** = 4, **σ** = 6.29) and the best prediction of the model without restrictions (**N** = 29, **σ** = 5.84)

It is possible that instead of analyzing all input colors (as was encouraged) and building the feature distribution representation, observers selected a small subset of disks for estimation using working memory and extrapolated this to the whole display. Such sampling has been suggested as an alternative explanation for previous explicit ensemble effects (Allik et al., 2013; Myczek & Simons, 2008; Whitney & Yamanashi Leib, 2018). Our main argument against this is that the FFR task used here is much harder than simply “report the average” because it requires obtaining a large amount of information for successful performance: what colors were present/not present, the relative frequency of all presented colors (sometimes very similar to each other). The use of this strategy is unlikely given capacity limits in working memory (three to four objects; Cowan, 2001; Luck & Vogel, 1997). However, to formally rule out this explanation, we developed a computational model that simulates performance based on this alternative strategy (Fig. 4A). The sub-sampling model receives all colors as input, adds perceptual noise to each of them (a random value from a Gaussian distribution with a mean equal to 0 and sd equal to σ, the first free parameter), and selects ***N*** disks (the second free parameter). Subsequently, it counts the number of disks in this sub-sample with the same color as the tested one,^1^ and to account for the number of objects in the full display, this number is multiplied by a random integer^2^ ranging from 1 to 36/***N.*** This rounded number is the response in a trial.

We fitted the model to the observer's individual data using a maximum likelihood approach. The performance of all participants was best explained by a large number of sampled objects (***N*** ranging from 19 to 36) and a high level of perceptual noise (Fig. 4B). Table 1 shows the best parameter fits for each observer. Even the lower bound of the sample size range far exceeds all realistic estimates of working memory capacity. Therefore, the performance levels of all participants cannot be achieved by sub-sampling a few disks. Figure 4C illustrates this by depicting all responses of one observer in the Gaussian condition along with the best prediction of the model with the restriction of sampling only four objects. Importantly, we do not argue that observers can hold 20 + objects in their working memory and perform counting operations on them, but instead that observers integrated information from the object ensemble (see *Discussion*).

**Table 1:** Best fits of the sub-sampling model for each participant

Observer #	Sample size, ***N***	Noise, σ
1	36.00	4.94
2	25.00	4.40
3	25.00	4.35
4	30.00	5.89
5	26.00	4.61
6	31.00	6.54
7	30.00	4.00
8	19.00	3.44
9	33.00	6.67
10	36.00	7.91

**Fig. 5:**
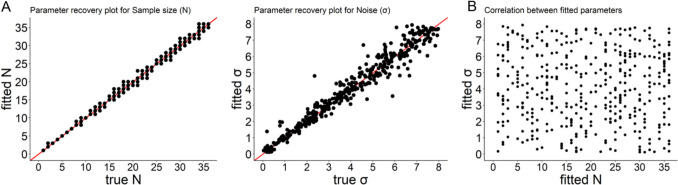
(**A**) Parameter recovery plots for sample size and noise. The parameter recovery plot for Sample size shows fewer than 400 dots due to the integer nature of this parameter – different dots can fully overlap. (**B**) No correlation between fitted values of sample size and noise

To provide evidence for the reliability of these estimates, we ran a parameter recovery procedure. We simulated the model responses using 400 random pairs of parameter values and fit this data using the same maximum likelihood approach to recover the estimated parameter values. The parameter recovery plots (Fig. 5) show excellent recovery for both parameters (*r*s > 0.97, *p*s < 0.001), meaning that the fitting procedure is quite precise. Importantly, the fitted parameters do not correlate with each other (*r* = −0.009, *p* = 0.86).

## Discussion

What does our conscious representation of object ensembles consist of? The *Summary statistics* view states that the visual system explicitly represents only their mean and variance. Here, we show for the first time that this *significantly underestimates* the richness of *explicit* ensemble representations. After brief exposure to a color ensemble, observers can accurately report the frequency of any color in the set. This means that they have explicit access not only to the mean color and variance of a set but also the relative frequency of each color – i.e., to the whole feature distribution. Our results, therefore, necessitate a fundamental rethinking of how visual ensembles are processed.

To build this distributional ensemble representation, the visual system must use information from all or most objects within the set. Our modeling shows that performance cannot be explained by sub-sampling unless observers can hold 20–35 objects in working memory and perform precise counting operations on them. Even though our task can be considered a variation of numerosity reports (Burr & Ross, 2008), it is highly unlikely that numerosity systems explain our results since the maximum number of subsets observers can successfully enumerate in parallel is three (Halberda et al., 2006) while the FFR task requires keeping track of nine subsets.

The population response model of ensemble perception (Utochkin et al., 2024) provides a parsimonious account of our findings. Each object is coded by a population of feature-selective neurons from lower levels of the visual hierarchy with small receptive fields. At later stages, feature-selective neuronal populations with larger receptive fields pool lower-level information, representing it as a distribution of the ensemble features without spatial details. This rich distribution representation can be explained without postulating new cognitive mechanisms but as the combined work of two well-known neural mechanisms: population coding and spatial pooling. Our results provide clear evidence for such representations and demonstrate, for the first time, that observers have explicit access to them.

Our *Feature distribution *view also suggests that summary statistics representations are not the core units of ensemble perception as was previously assumed (Alvarez, 2011; Ariely, 2001; Chong & Treisman, 2003, 2005b, 2005a; Haberman et al., 2015) but rather derivatives of explicit feature distribution representations. There is no substantial difference between the representations in our FFR task and classical “report the average/variance” tasks. In both cases, the visual system represents the whole feature distribution but performs operations appropriate for the requsted response. In the FFR task, observers access specific parts of the feature distribution, while during the average report, they can report the feature distribution peak.

The *Feature distribution* view entails a shift in the focus of ensemble perception studies. The current definitions of ensemble representations refer to “extracted statistical summaries of sets” (Corbett et al., 2023) or “statistical moments” (Whitney & Yamanashi Leib, 2018). Adopting the *Feature distribution* view requires studying the distributional representation that summary representations are derived from. The mushroom-fungus metaphor illustrates this. One way to understand how a specific fungus (e.g., champignons) functions is to study its mushrooms. But the mushrooms are only an above-ground manifestation of the fungus that lies underground. Similarly, we argue that understanding how the visual system represents sets of objects (“fungi”) requires studying representations of feature distributions instead of focusing on specific operations (extracting the summary statistics) that can be performed with this representation (“mushrooms”). The new Feature Frequency Report paradigm enables the investigation of explicit representations of feature distributions using a simple task that requires neither explicit questions about distributional shapes nor reproductions of them (e.g., Oriet & Hozempa, 2016). The FFR paradigm also does not require the comparison of different visual displays, sidestepping the problem of keeping all statistical characteristics stable except the studied one (Atchley & Andersen, 1995).

Most previous evidence for detailed feature distribution representations has come from indirect measures, such as repeated visual search (Feature Distribution Learning paradigm), leading to speculations that these representations are mostly implicit (Chetverikov & Kristjánsson, 2022; Chetverikov et al., 2016, 2017b; Hansmann-Roth et al., 2021). This raised questions about the functional relationship between implicit and explicit distribution representations: Are they the same representation or two different ones, originating from partially non-overlapping cognitive/neural mechanisms? Using the Feature Distribution Learning paradigm combined with implicit and explicit probe tasks, Hansmann-Roth and colleagues (2021) found evidence for the implicit but not explicit representation of feature distributions. They also observed no correlation between individual performance in implicit and explicit tasks. They suggested that implicit and explicit ensemble representations can originate through different pathways. However, our newly developed paradigm shows that the visual system builds *explicitly* available distributional ensemble representations. These results suggest that explicit and implicit representations originate from a common pathway, a view that is grounded in an established computational framework (Utochkin et al., 2024). However, the present study does not assess implicit representation nor the correlation between performance on implicit and explicit tasks. We, therefore, cannot fully resolve this debate. For now, we note the important differences between the two sets of findings. First, implicit representations arise automatically as a byproduct of another task (visual search), and observers are unaware that their representations of distractor distributions are being probed. Conversely, in our explicit-report paradigm, observers know that they should report the frequency of a random color. The role of attention in building explicit representations, therefore, needs to be assessed. Second, implicit representations require multiple exposures to the distributions: at least 2 for Gaussian and uniform distributions and 7–10 for more complicated distributions such as bimodal ones (Chetverikov et al., 2017a, 2020). In contrast, we show here that reliable explicit distributional representations can form after only a single exposure (including bimodal distributions). Additionally, Khayat and colleagues (2024) showed that explicit ensemble representations in several “report average” tasks were more precise than implicit ones.

There is a discrepancy between our seemingly rich perceptual experience and the objective limitations of our attention and working memory systems (Cowan, 2001; Luck & Vogel, 1997; Pylyshyn & Storm, 1988; Rensink et al., 1997). Ensemble summaries are thought to compensate for this (Cohen et al., 2016): people can only attend to and process a few objects at a time while other information is encoded as summaries. We suggest that this feeling of rich perceptual experience can originate not from summary statistics of multiple objects but from explicit representations of feature distributions. While people do indeed have detailed representations of many objects, precise spatial information may be lacking. Thus, despite providing rich perceptual experience, these representations may be less helpful in paradigms requiring spatial information for successful performance, such as change detection (Luck & Vogel, 1997) or change blindness (Rensink et al., 1997).

## Conclusion

We demonstrate that the visual system can represent whole feature distributions and, most importantly, that observers have reliable explicit access to this representation. This finding contradicts the popular view that observers have explicit access only to representations of summary statistics. We propose the *Feature distribution* view of ensemble perception: when the visual system perceives a set of objects, the feature distribution is represented in detail. This representation is explicitly accessible and can be used to report distribution characteristics and summary statistics. Observers consciously represent stimulus ensembles as a feature distribution, not as a limited set of summary statistics.

## Authorsʼ contributions

V.K. designed and performed the experiments, derived the model, analysed the data, and wrote the first version of the manuscript. A.G.A. and A.K. contributed to the final version of the manuscript, supervised the project at all stages, and obtained funding.

## Data Availability

All study materials and data are publicly available via the Open Science Framework at: https://osf.io/a76w4/

## Footnotes

1 To be precise, the model counts the number of disks whose color values (after adding the perceptual noise) are closer to the tested color value compared to any other presented value. For example, the tested color value was 3 (JND from the mean), a disk with 4.26 value will be counted however a disk with 4.59 will not (since it is closer to another presented color value – 6 JND).

2 We also made a simpler model that follows the same steps, except it always multiplies the number of counted disks by 36/N (which is more mathematically accurate). We do not present it here because, for small sample sizes, it produces a non-realistic set of responses consisting only of zeros and eights. Nevertheless, the fits are similar to the presented model.

## Code availability

Experimental and analysis scripts are publicly via the Open Science Framework at: https://osf.io/a76w4/
